# A Chinese family with congenital Dysfibrinogenemia carries a heterozygous missense mutation in FGA: Concerning the genetic abnormality and clinical treatment

**DOI:** 10.12669/pjms.334.12828

**Published:** 2017

**Authors:** Jihao Zhou, Peng Zhu, Xinyou Zhang

**Affiliations:** 1 Jihao Zhou, M.D. Department of Hematology, The Second Medical College (Shenzhen People’s Hospital), Jinan University, Shenzhen, Guangdong Province, P.R. China; 2 Peng Zhu, M.D. Key Laboratory, The Second Medical College (Shenzhen People’s Hospital), Jinan University, Shenzhen, Guangdong Province, P.R. China; 3 Xinyou Zhang, M.D. Department of Hematology, The Second Medical College (Shenzhen People’s Hospital), Jinan University, Shenzhen, Guangdong Province, P.R. China

**Keywords:** Dysfibrinogenemia, Fibrinogen replacement treatment, Mutation, Pulmonary embolism

## Abstract

**Objectives:**

Congenital dysfibrinogenemia is a rare hereditary disease characterized by normal antigen level but lower function level of fibrinogen. Patients with congenital dysfibrinogenemia usually present as bleeding and/or thrombotic events. In this study, we explored the genetic abnormality and clinical treatment of a Chinese family with dysfibrinogenemia.

**Methods:**

This study was conducted in Jan 2015 to Jan 2016 in the Second Medical College (Shenzhen People’s Hospital, Jinan University, Shenzhen, Guangdong Province, P.R. China. Coagulation function test were used to screen patients in this family. For all family members, DNA from peripheral blood was isolated. Whole-genome exon sequencing was carried out to screen possible mutations. And sanger sequencing was employed to further confirm the mutation in patients.

**Results:**

The proband is a woman who had anemia and increased menstruation. Hypofibrinogenemia was found after admission. However, a pulmonary embolism occurred after the fibrinogen replacement treatment. Whole exon sequencing was conducted afterward. A candidate mutation in FGA gene (c.103C>A) was identified and validated in the woman and in two siblings.

**Conclusion:**

From this case, we learned that1) point mutation of c.103C>A is the pathogenesis for congenital dysfibrinogemia in this family; 2) thromboprophylaxis should always be in consideration when fibrinogen replacement is conducted. Prospective studies are needed to determine the best fibrinogen replacement strategy in order to achieve adequate hemostasis while minimize risk of thrombosis.

## INTRODUCTION

Congenital dysfibrinogenemia is a rare hereditary disease characterized by normal antigen level but lower function level of fibrinogen. Patients with congenital dysfibrinogenemia usually present as bleeding and/or thrombotic events.^1^ The major treatment for this disease, especially when patients experience bleeding, is fibrinogen replacement therapy. However, such a replacement strategy may be associated with an increased risk of thrombotic events, which constitute a clinical dilemma.^2^ In this article, we have reported a congenital dysfibrinogenemia patient who suffered from pulmonary embolism after fibrinogen replacement treatment. Our goal was to identify the disease-causing mutation of this family, and try to review current knowledge about treatment for these patients.

## METHODS

This study was conducted in Jan 2015 to Jan 2016 in the Second Medical College (Shenzhen People’s Hospital, Jinan University, Shenzhen, Guangdong Province, P.R. China We performed routine coagulation function test including prothrombin time, activated partial thromboplastin time, thrombin time, and the level of fibrinogen for all donors and family members enrolled. Von Clauss method was used for the screening test for fibrinogen. However, the fibrinogen antigen levels were tested for confirmation for patients whose fibrinogen activity level was low.

### DNA isolation and whole-genome exome sequencing

DNA for genetic analysis from family members and all donors were isolated from peripheral blood using DNA isolation kit (Qiagen, Hilden, Germany). The genomic DNA from the proband was fragmented and enriched. The whole-genome exome sequencing (WES) was carried out at Huada Gene Research Center (Shenzhen, China).

### Sanger sequencing

Primer sequences for the specific coding region were designed by using a public primer design website. Direct Sanger sequencing using primers flanking the mutated exome of the target gene was used to validate the identified mutation. Once the mutation in the proband was validated, Sanger sequencing was performed to all other family members and healthy donors enrolled.

## RESULTS

A Chinese Han family featuring low level of fibrinogen was clinically examined. The family members available consist of four siblings, and their five healthy sons and daughters. Another 100 healthy donors were also enrolled as controls. Written informed consent was obtained from all donors and the family members. All our experiments were approved by the ethics committee of the Second Medical College (Shenzhen People’s Hospital), Jinan University. The proband was a 51-year-old female. She was admitted to our hematology department in Jan, 2015, with a main complaint of fatigue and dizziness for about 20 days. Twenty days before admission, the patient felt fatigue and dizziness, with palpitation after mild exercise. No fever, dyspnea, jaundice, purpura or osteodynia was complained, but the symptoms aggravated. The patient denied soy urine and melena. However, she found significantly increased menstruation in recent three months, which lasted for more than 10 days each cycle. Her current menstrual cycle had lasted for more than two weeks until her admission to our hospital. Her medical history was negative, except for a history of cesarean birth. No massive hemorrhage was reported in her life, even when she was pregnant or in the delivery process. She denied history of any hereditary diseases in her family. Physical examination revealed nothing special except an obvious anemic appearance. No bleeding on the skin or the mucosa was observed. After admission, a CBC revealed a microcytic hypochromic anemia, with a WBC count of 8.4×10^9^/L, a HGB level of 60g/L, a MCV of 71fl, a MCH of 27pg/L, and a PLT count of 397×10^9^/L. A routine coagulation test showed a normal PT of 14.3s (11.4s), a normal APTT of 24.3s (24.8s), but a longer TT of 28.5s (18.2s) and a lower fibrinogen activity of 0.68g/L (tested by Von Clauss method). The ferritin level was 7.1ng/ml (the normal range was 11ng/ml). Therefore, the patient was diagnosed as hypofibrinogenemia and iron deficient anemia. At that time, the cause of anemia was contributed to her increased menstruation; however, the cause of her hypofibrinogenemia was unknown. Since the patient continued bleeding, she received 400ml fresh frozen plasma (FFP) and 10U cryoprecipitate for fibrinogen replacement treatment in the first night of her admission. But six hours after the transfusion finished, the patient suddenly felt cough, chest pain and dyspnea. She cannot lie down because of difficult breathing. Oxygen inhalation was given but showed little help.

**Fig. 1:**
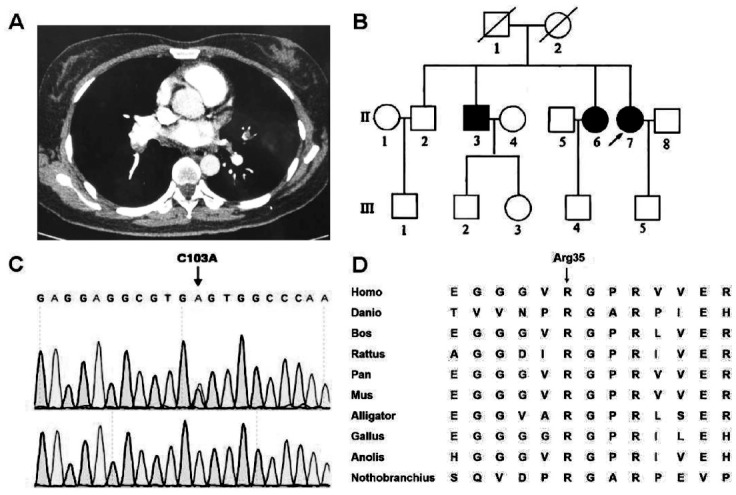
The clinical and genetic features of the patients. A) CTPA of the proband showed pulmonary embolism; B) The pedigree of the proband (arrow) and her family. The circles denote female family members; squares denote male family members. Solid symbols illustrate affected family members. Slash symbols illustrate dead family members; C) The result of sanger sequencing validated the heterozygous c.103C>A mutation in proband; D) Alignment of Arg(R)35 residue in FGA protein of other species indicates that this site is highly conservative among species.

A blood-gas analysis was immediately ordered. The result showed type I respiratory failure with an oxygen saturation of only 90%, in spite of an oxygen flow rate of 5L/min. D-dimer level was 5263.41ng/ml then. An emergent CTPA was conducted and confirmed massive pulmonary embolism of both pulmonary arteries, as is shown in Fig. 1A. Color Doppler Ultrasonography revealed intermuscular venous thrombosis of both calf, but found no thrombosis of both deep veins. The patient was then transferred to ICU and received low molecular weight heparin (LMWH) injection as anti-coagulation therapy. Considering that the patient also had a massive hemorrhage in the form of menstruation, FFP and cryoprecipitate were also administered at the same time of anti-coagulation therapy to keep a fibrinogen activity level more than 1.0g/L. After these measures, both of the patient’s symptoms of dyspnea and bleeding relieved, and she discharged 23 days after admission. Her fibrinogen activity level was 0.58g/L when she was discharged. She kept receiving LMWH injection for another 2 months.

The anti-coagulation treatment was continued until a CTPA examination was repeated and showed negative results. To investigate the cause of her hypofibrinogenemia further, we tested the patient’s fibrinogen antigen level, which was near normal (1.89 g/L). This discrepancy between fibrinogen antigen level and activity level indicated a diagnosis of dysfibrinogenemia. We screened the coagulation function of her family members available. We found one older sister and one older brother who also showed dysfibrinogenemia (both exhibited a normal fibrinogen antigen level, but a lower fibrinogen activity level by Von Clauss method) but reported no symptoms of hemorrhage or thrombotic events. However, none of their sons or daughters showed obvious coagulation abnormalities by routine coagulation test, as is shown in Fig. 1B.

### Genetic analysis result

After whole exome sequencing, a candidate heterozygous mutation in FGA gene was identified, and subsequently validated by sanger sequencing. The three affected family members all carry the specific mutation of a change from cytimidine to adenine at complementary DNA (cDNA) nucleotide 103 (c.103C>A), leading to the substitution of Arginine to Serine at residue 35 of FGA protein (Fig. 1C). Primers used for Sanger sequencing are designed as TCTCATAACAACTCCATAAAATGGG for the forward and GGAGGTAAATAAACAGTGCTCTTGG for the reverse. As is shown in Fig. 1D, Fig. 1 This residue of FGA protein is highly conservative among species. All affected family members were heterozygous, indicating that this mutation is autosomal dominant inherited. None of the 100 donors enrolled carried this mutation. By searching the database of 1000 genomes projects, polymorphism was further excluded. Therefore, we concluded that this mutation is the pathogenic mutation for affected members in this family.

## DISCUSSION

Congenital dysfibrinogenemia is a hereditary disease characterized by qualitative fibrinogen deficiency, in spite of relatively normal quantity of serum fibrinogen antigen level. The first patient was reported by Imperato in 1958.^3^ Then in 1968, the first point mutation associated with this disease was identified.^4^ Currently, the pathogenesis of congenital dysfibrinogenemia is contributed to mutations in the three genes which encode fibrinogen together, that are genes FGA, FGB and FGG. More than 100 mutations in the three genes have already been identified and kept update.^5^ Some of these mutations affect fibrinolysis, some affect fibrinogen polymerization, some affect fibrinogen-fibrin structure, and some remain unknown. According to fibrinogen mutation database, the mutation we found in this family (FGA Arg35Ser) has been reported by other researchers in 2013^6^ and in 2015^7^ respectively. Patients carrying this mutation were all reported as bleeding discrepancy without thrombotic complications. Although no function characteristics has been associated with this specific mutation, there were studies saying that Arg35 is the most frequently mutated residues, or the so-called “hot-spot mutation point” in fibrinogen genes.^8^,^9^ Since FGA Arg35 is part of the thrombin cleavage site at the N-terminal end of the fibrinogen A α chain, mutation at this site was predicted to result in delayed or absent fibrin peptide A release and subsequent prolonged polymerization.^10^,^11^ Besides, there are also studies reported that FGA Arg35 mutation might affect strength of fibrin network^12^, induce fibrinolysis resistance^13^, or decrease platelet aggregation support.^14^ All the speculated possible function mutation related with FGA Arg35 mutation are reasonably related to a clinical manifestation of bleeding discrepancy. However, the patient in our study suffered not only a prolonged menstruation bleeding, but also a lethal massive pulmonary embolism. How a thrombotic event could occur in such a patient prone to bleeding? This patient’s paradoxical clinical manifestation puzzled us. So we reviewed literatures discussing pulmonary embolism in hypofibrinogenemia patients.

After literature review, we found that although this patient we reported is rare, she is not exceptional. A total of 17 similar cases were reported before. Those patients’ clinical data have already been reviewed by Girolami et al. in 2016.^15^ Among those patients combined with hypofibrinogenemia and pulmonary embolism, 47% (8/17) patients were diagnosed as afibrinogenemia, 47% (8/17) were diagnosed as dysfibrinogenemia, while 6% (1/17) patient was diagnosed as hypofibrinogenemia without classification. The average age was 33.4(14 the youngest and 61 the oldest) years old. Seven cases were male. The other cases were female. Among those patients, 7 out of 17 received fibrinogen replacement treatment before their occurrence of pulmonary embolism. The reviewer concluded that fibrinogen replacement treatment is one of the main risk factors for pulmonary embolism in these patients. However, in consideration that most hypofibrinogenemia patients who received fibrinogen replacement treatment are combined with hemorrhage, what is the best replacement strategy remains unknown. Currently, only a guideline based on experts’ opinion from the UK Haemaphilia Center Doctors was first published in 2004 and then updated in 2014.^16^,^17^ This guideline provided practical instructions about fibrinogen replacement in dysfibrinogenemia patients, including:

1)For mild hemorrhage or minor surgery, consider tranexamic acid 15-20mg/kg or 1g four times daily alone
2)For severe bleeding or major surgery, consider fibrinogen concentrate 50-100mg/kg, with smaller dose repeated in necessary at 2-4 days’ interval to maintain fibrinogen activity over 1g/L
3)For patients with risk factors to thrombosis, consider thromboprophylaxis with LMWH.
4)Considering cryoprecipitate if fibrinogen concentrate is unavailable.

In 2016, a consensus about Management of congenital quantitative fibrinogen disorders achieved among invited haemophilia experts from Belgium, France and Switzerland was also published.^18^ Even though the type, dose and frequency of fibrinogen replacement treatment were suggested, venous or arterial thrombosis still occurred in 30% patients in the case report series.^2^,^19^ Some researchers have suggested that thromboelastography may predict risks of thrombosis complication occurrence in (hypo) dysfibrinogenemia patients.^20^ Such a method could be a helpful tool for physicians to decide whether fibrinogen replacement is required, however, more studies are needed to further explore its diagnostic value.

## CONCLUSION

In conclusion, there is no commonly accepted treatment standard for fibrinogen replacement treatment for congenital dysfibrinogenemia patients. From the case we reported and from literatures we reviewed, we suggest that thromboprophylaxis should always be in consideration when fibrinogen replacement is conducted. And the most important thing we can do in dealing with these patients may be closely monitoring of fibrinogen activity and D-dimer after fibrinogen replacement to balance the risk between hemorrhage and thrombosis. Prospective studies are needed to determine the best fibrinogen replacement strategy in order to achieve adequate hemostasis while minimize risk of thrombosis.

### Authors’ Contributions

**XZ** designed this study.

**JZ** collected, analyzed the data and prepared this manuscript.

**PZ** performed the whole-genome exon sequencing.
